# Decreased TLR7 expression was associated with airway eosinophilic inflammation and lung function in asthma: evidence from machine learning approaches and experimental validation

**DOI:** 10.1186/s40001-023-01622-5

**Published:** 2024-02-10

**Authors:** Kemin Yan, Yuxia Liang

**Affiliations:** 1 Department of Geriatrics, The First Affiliated Hospital of Sun Yat-Sen University, Guangzhou, Guangdong China; 2 Department of Pulmonary and Critical Care Medicine, The First Affiliated Hospital of Zhengzhou University, Zhengzhou, China; 3 Department of Pulmonary and Critical Care Medicine, The First Affiliated Hospital of Sun Yat-Sen University, Guangzhou, Guangdong China

**Keywords:** Asthma, TLR7, Induced sputum, Machine learning, Immune cell infiltration

## Abstract

**Background:**

Asthma is a global public health concern. The underlying pathogenetic mechanisms of asthma were poorly understood. This study aims to explore potential biomarkers associated with asthma and analyze the pathological role of immune cell infiltration in the disease.

**Methods:**

The gene expression profiles of induced sputum were obtained from Gene Expression Omnibus datasets (GSE76262 and GSE137268) and were combined for analysis. Toll-like receptor 7 (TLR7) was identified as the core gene by the intersection of two different machine learning algorithms, namely, least absolute shrinkage and selector operation (LASSO) regression and support vector machine-recursive feature elimination (SVM-RFE), and the top 10 core networks based on Cytohubba. CIBERSORT algorithm was used to analyze the difference of immune cell infiltration between asthma and healthy control groups. Finally, the expression level of TLR7 was validated in induced sputum samples of patients with asthma.

**Results:**

A total of 320 differential expression genes between the asthma and healthy control groups were screened, including 184 upregulated genes and 136 downregulated genes. TLR7 was identified as the core gene after combining the results of LASSO regression, SVM-RFE algorithm, and top 10 hub genes. Significant differences were observed in the distribution of 13 out of 22 infiltrating immune cells in asthma. TLR7 was found to be closely related to the level of several infiltrating immune cells. TLR7 mRNA levels were downregulated in asthmatic patients compared with healthy controls (*p* = 0.0049). The area under the curve of TLR7 for the diagnosis of asthma was 0.7674 (95% CI 0.631–0.904, *p* = 0.006). Moreover, TLR7 mRNA levels were negatively correlated with exhaled nitric oxide fraction (*r* = − 0.3268, *p* = 0.0347) and the percentage of peripheral blood eosinophils (%) (*r *= − 0.3472, *p* = 0.041), and positively correlated with forced expiratory volume in the first second (FEV1) (% predicted) (*r* = 0.3960, *p* = 0.0071) and FEV_1_/forced vital capacity (*r* = 0.3213, *p* = 0.0314) in asthmatic patients.

**Conclusions:**

Decreased TLR7 in the induced sputum of eosinophilic asthmatic patients was involved in immune cell infiltration and airway inflammation, which may serve as a new biomarker for the diagnosis of eosinophilic asthma.

**Supplementary Information:**

The online version contains supplementary material available at 10.1186/s40001-023-01622-5.

## Background

Asthma is a common chronic disease in which airways become inflamed and narrow, causing airflow obstruction [1–3]. Asthma is a heterogeneous clinical syndrome that affects more than 300 million people worldwide [4]. The common symptoms of asthma in the acute phase include wheezing, coughing, chest tightness, and shortness of breath [1, 2]. Asthma is a complex and heterogenous respiratory diseases. The underlying pathogenetic mechanisms of asthma were poorly understood [5].

Induced sputum has several desirable characteristics as a noninvasive marker of airway inflammation [6]. In patients with asthma, sputum induction is generally a well-tolerated and safe method, and sputum can be used to measure various soluble mediators, including eosinophilic-derived proteins, cytokines, and remodeling-related proteins [6–11]. Induced sputum may be used to discover inflammatory cell profiles in patients with asthma and other airway diseases, and these profiles may be related to the patient’s response to treatment [12]. The gene expression profile of induced sputum cells is altered in patients with asthma [13].

Microarray technology and integrated bioinformatics analysis have been used in recent years to identify novel genes associated with various diseases that may serve as biomarkers for diagnosis and prognosis [14, 15]. Bioinformatics analysis has also been performed to identify the underlying mechanisms and hub genes of asthma [16, 17]. Studies have also shown that immune cell infiltration plays an increasingly important role in the occurrence and development of various diseases [18–21]. Previous studies demonstrated that the Th1/Th2-mediated immune imbalance is the main mechanism of asthmatic airway inflammatory response, and various immune cells are involved in the pathogenesis of asthma [22].

CIBERSORT, a method for characterizing cell composition of complex tissues from their gene expression profiles, has been widely used to evaluate the relative content of 22 kinds of immune cells [23]. CIBERSORT method has also been applied to study the immune cell infiltration and candidate diagnostic markers in asthma. It has been reported by Yang et al. that autophagy-related genes are involved in the progression and prognosis of asthma and regulate the immune microenvironment [24]. Least absolute shrinkage and selector operation (LASSO) regression and support vector machine-recursive feature elimination (SVM-RFE) are two machine learning algorithms. LASSO is a dimension-reduction algorithm that can analyze high-dimensional data compared with regression analysis [25]. SVM-RFE is a machine learning algorithm used to identify the best variables through classification method [26]. The combination of LASSO and SVM-RFE has been applied in previous research to identify diagnostic markers [20, 27, 28].

In the present study, bioinformatics analysis and experimental validation were performed to investigate the change of immune cell infiltration in asthma, and screen the biomarker for the diagnosis and treatment of asthma. Two datasets from Gene Expression Omnibus (GEO) database were combined, and differential expression gene (DEG) analysis, machine learning algorithms and CIBERSORT were performed. Toll-like receptor 7 (TLR7), a candidate gene that was found to be closely associated with immune infiltration in asthma, was also validated in another GEO dataset and induced sputum samples of asthmatic patients.

## Material and methods

### Subjects

We recruited 12 healthy controls and 36 newly diagnosed asthma patients with untreated asthma. The asthmatic patients included in this study and the control group were non-smokers, and the asthmatic patients were newly diagnosed and untreated. The asthmatic patients were from outpatients and were diagnosed with asthma by specialists. The characteristics of the subjects are summarized in Table 1. No significant differences were observed in terms of age, sex, and body mass index between the two groups. All subjects provided written informed consent. The study was approved by the Ethics Committee of the First Affiliated Hospital of Sun Yat-sen University (2021071).

**Table 1:** Characteristics of subjects

	Healthy controls	Asthma	*p* value
Number	12	36	
Sex, F:M (%F)	8/4 (66.67)	13/23 (36.11)	0.0951
Age, yr	35.75 ± 15.58	44.056 ± 16.81	0.1331
BMI, kg/m2	23.278 ± 3.8964	23.098 ± 3.38	0.8611
Lung function			
FEV1, % predicted	93.5 (90–108)	88.89 (61.65–101.69)	0.1739
FEV1/FVC%	87 (81.4–90.91)	74 (59.93–78.44)	< 0.0001
FeNO, ppb	11 (9–14)	38 (31–66)	< 0.0001
Blood-eosinophil, %	1.95 (1.125–3.35)	4.6 (2.3–7.6)	0.0236

Values are presented as mean ± SD or median (interquartile spacing)

*FeNO* fraction of exhaled nitric oxide, *FEV1* forced expiratory volume in the first second, *FVC* forced vital capacity

### Dataset acquisition and processing

The study design is shown in Additional file 1: Fig S1. The datasets GSE76262 and GSE137268 were downloaded from the GEO database (http://www.ncbi.nlm.nih.gov/geo). GSE76262 dataset, which is based on GPL13158 platform, included induced sputum samples from 118 asthmatic patients and 21 healthy controls. GSE137268 dataset, which is based on GPL6104 platform, included induced sputum samples from 54 asthmatic patients and 15 healthy controls. The series matrix files were annotated to the official gene symbols, and the two gene expression files were merged. The batch normalization was then conducted using combat method in “sva” R package. Finally, a merged file with 15,043 genes was prepared for the subsequent analysis.

### Identification of DEGs and enrichment analysis

The “limma” R package was used to identify DEGs, and the |log2FC|> 0.5 and adjusted *p* value < 0.05 were filtered as statistically significant. Gene Ontology (GO) and Kyoto Encyclopedia of Genes and Genomes (KEGG) analyses were then performed using “clusterProfiler” R package. Gene Set Enrichment Analysis (GSEA) was conducted to analyze the associated biological functions and pathways in asthma. Disease Ontology (DO) was also conducted using “DOSE” R package.

### Identification of the core gene

First, two distinct machine learning algorithms, namely, least absolute shrinkage and selector operation (LASSO) regression and support vector machine-recursive feature elimination (SVM-RFE), were utilized in DEGs to screen the gene signatures. The LASSO is a regression analysis algorithm that uses regularization to improve the prediction accuracy. The LASSO analysis was undertaken using “glmnet” R package; the response type was set as binomial, and the alpha was set as 1. SVM is a supervised machine-learning technique widely utilized for both classification and regression. To avoid overfitting, an RFE algorithm was employed to select the optimal genes from the meta-data cohort. Therefore, to identify the set of genes with the highest discriminative power, SVM-RFE was applied to select the appropriate features. The SVM-RFE was performed using “e1071” and “caret” R package. Second, STRING database was used to construct the protein–protein interaction (PPI) network, and a core network was obtained through Cytoscape software and CytoHubba plugin. The top 10 hub genes were screened according to Degree algorithm. Finally, the results of LASSO regression, SVM-RFE algorithm, and hub genes were incorporated, and the overlapping gene (TLR7) was identified as the core gene.

### Analysis of immune cell infiltration

The CIBERSORT algorithm was used to evaluate the percentage of 22 immune cell types in each sample. The fraction of 22 immune cells was compared between the asthma and healthy control groups, and the violin plot was drawn by “vioplot” R package. The correlation coefficient between immune cells was calculated using “corrplot” R package. Spearman correlation analysis was also performed to investigate the correlation of TLR7 and infiltrating immune cells.

### Validation of TLR7 in a GEO dataset

The expression level of TLR7 in the merge dataset was visualized, and receiver operating characteristic (ROC) curve was applied to evaluate the diagnostic value of TLR7 for asthma. Furthermore, the GEO dataset GSE147878 with endobronchial biopsy samples from 60 asthmatic patients and 13 healthy controls was used to validate the expression level and diagnostic effectiveness of TLR7 in asthma.

### Collection of induced sputum from subjects

A total of 48 subjects from First Affiliated Hospital of Sun Yat-sen University (Guangzhou, Guangdong, China) were enrolled in this study, including 12 healthy controls and 36 asthmatic patients. Patients with asthma met the diagnostic criteria for Global Asthma Initiative (GINA) guidelines [29] and were free of other respiratory diseases. People with normal lung function test results and no history of pulmonary disease, allergic disease, and autoimmune disease were included in the healthy control group. Sputum samples were collected from the participants. Participants were induced to cough by hypertonic saline. The above steps are completed by ultrasonic atomizer (Yuyue, Jiangsu, China). Sputum cell pellet was selected, weighed, and dissolved by adding 0.1% dithiothreitol (DTT) that is 4 times the weight. The pellet was then filtered through cell sieving [30–32]. After centrifugation, sputum cells were added with 1 ml TRIzol for subsequent RNA extraction. Additional clinical information was collected for each subject, including lung function, exhaled nitric oxide fraction (FeNO), and peripheral blood eosinophil percentage.

### Quantitative real‐time polymerase chain reaction (qRT-PCR)

Total RNA was extracted from induced sputum cells using TRIzol reagent following the manufacturer’s instructions. Evo M-MLV RT Premix kit (AG, Hunan, China) was used for reverse transcription. The reaction conditions were 37 ℃ for 15 min and 85 ℃ for 5 s. Candidate gene expression was quantified using Biosystems Light Cycler 480 (Applied Biosystems, Massachusetts, USA) as standard procedure. The primers used were TLR7: forward, 5′- TCCTTGGGGCTAGATGGTTTC-3′, reverse, 5′- TCCACGATCACATGGTTCTTTG-3′ and GAPDH: forward, 5ʹ-ACCCAGAAGACTGTGGATGG-3ʹ, reverse, 5ʹ-TTCTAGACGGCAGGTCAGGT-3ʹ.

### Statistical analysis

All data in this study were analyzed through GraphPad Prism 8. 0 (GraphPad, San Diego, California, USA). Normally distributed data were obtained through unpaired t-test and expressed as mean ± standard deviation. For non-normally distributed data, the results were obtained via a nonparametric test (i.e., Kruskal–Wallis test) and expressed as median (interquartile spacing). Fisher’s exact test was used to analyze classified data, and Spearman rank correlation was used for correlation analysis. ROC was generated to determine the diagnostic value of TLR7. *P* < 0.05 was considered statistically significant.

## Results

### Identification of DEGs and enrichment analysis

The inclusion criteria of the DEGs were |log2FC|> 0.5 and adjusted *p* value < 0.05. A total of 320 DEGs between the asthma and healthy control groups were screened, including 184 upregulated genes and 136 downregulated genes. The expressions of the DEGs in each sample are shown in the heatmap (Additional file 1: Fig S2A), and the distribution of the DEGs is illustrated through a volcano plot (Additional file 1: Fig S2B).

**Fig. 1:**
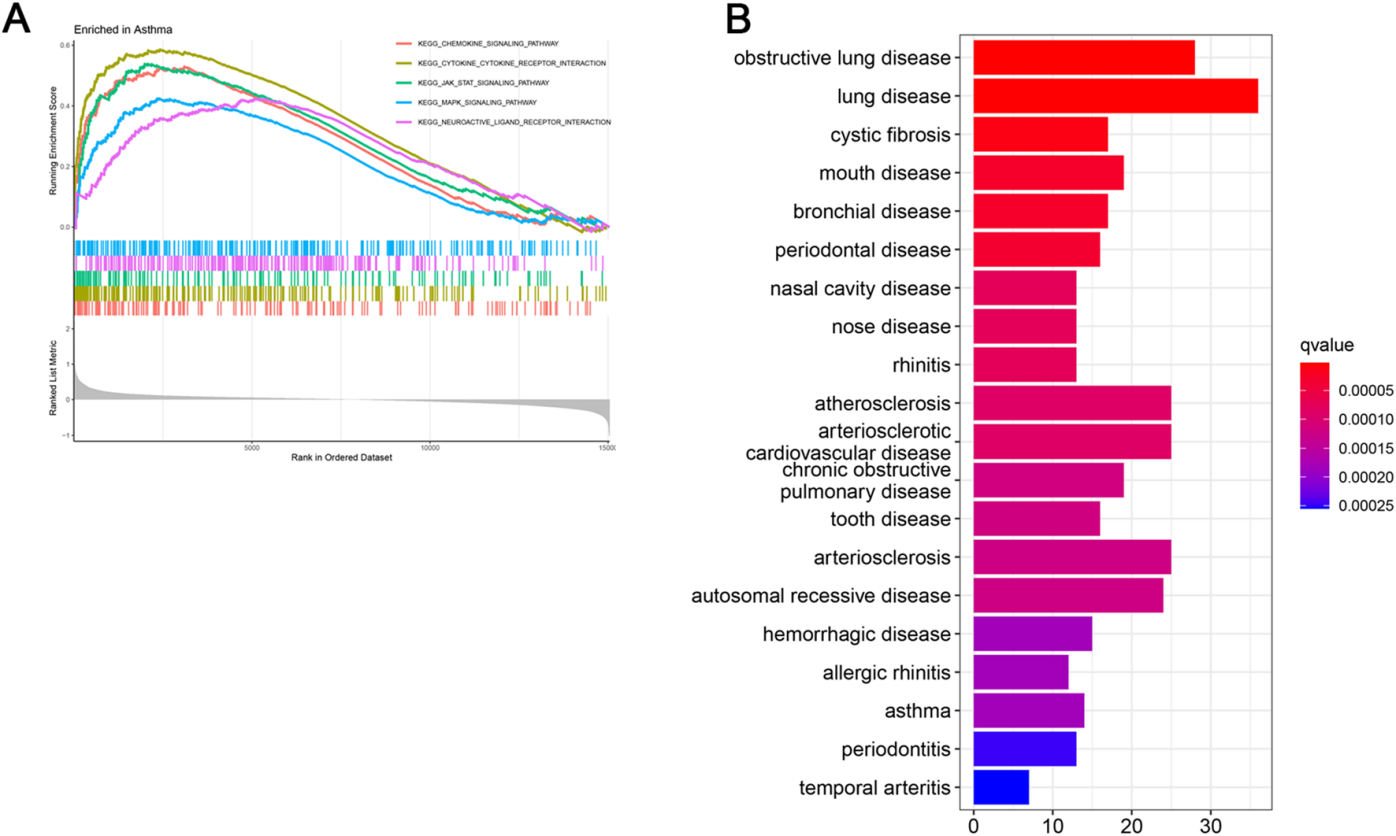
GSEA and DO enrichment analyses. **A** Illustration of several important enrichment hallmark terms in asthma obtained through GSEA. **B** Top 20 terms in the DO enrichment analysis

GO, KEGG, GSEA, and DO analyses were performed to further investigate the DEGs’ functions. GO enrichment analysis was conducted to analyze the gene function in terms of biological processes (BP), cellular component (CC), and molecular function (MF). The GO analysis results showed that BP is mainly enriched in the regulation of immune effector process, CC is mainly enriched in tertiary granule, and MF is mainly enriched in immune receptor activity (Additional file 1: Fig S3A). The KEGG pathway enrichment analysis demonstrated that the DEGs were mainly involved in cytokine–cytokine receptor interaction, tumor necrosis factor (TNF) signaling pathway, and nuclear factor (NF)-kappa B signaling pathway (Additional file 1: Fig S3B). As shown in Fig. 1A, GSEA also showed that the significantly enriched hallmark terms associated with asthma included chemokine signaling pathway, cytokine–cytokine receptor interaction, Janus kinase/signal transducer and activator of transcription (JAK/STAT) signaling pathway, mitogen-activated protein kinase (MAPK) signaling pathway, and neuroactive ligand receptor interaction. Furthermore, DO analysis revealed that the DEGs were mainly related to lung disease and obstructive lung disease (Fig. 1B).

### Identification of TLR7 as the core gene

**Fig. 2:**
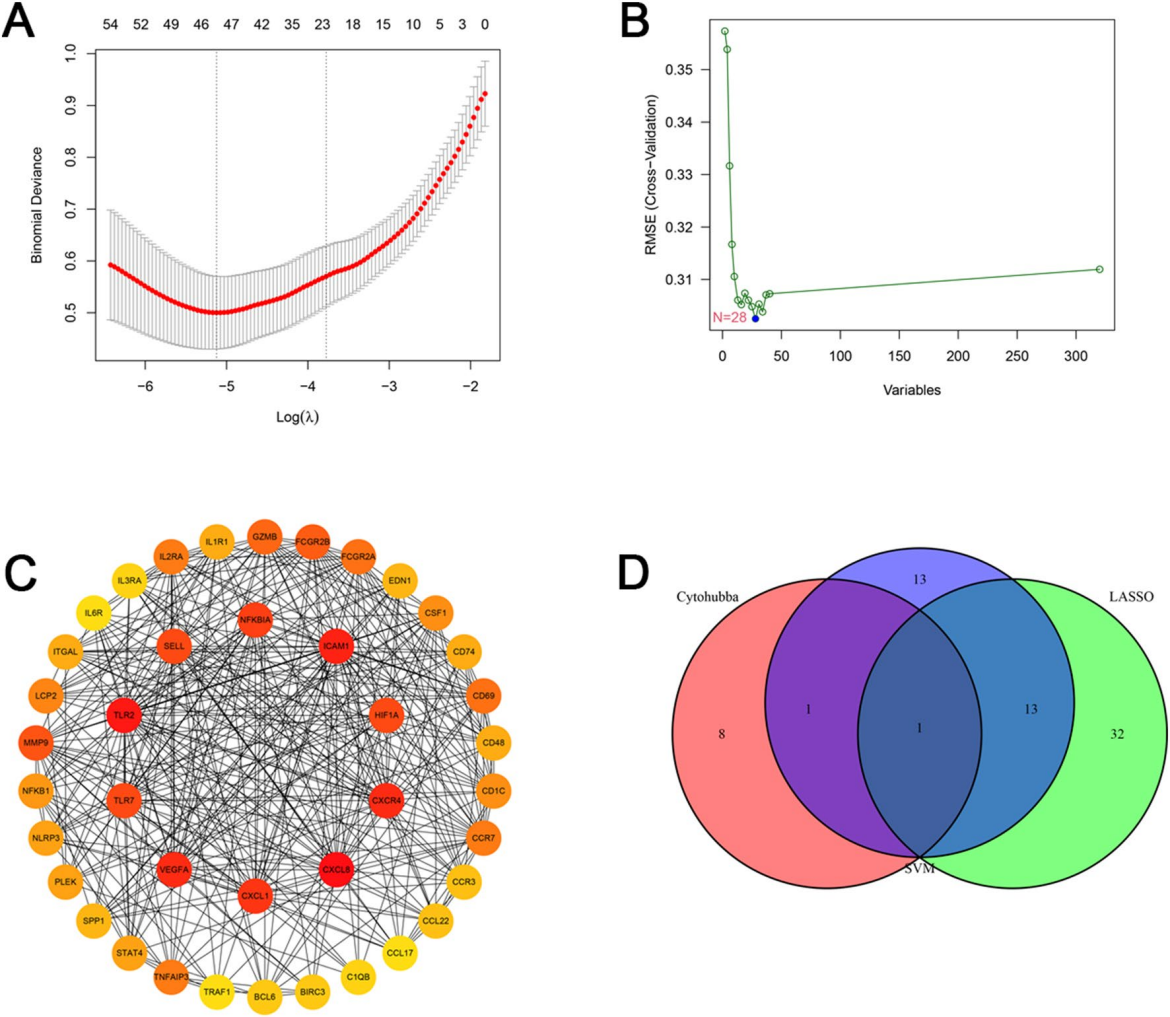
Two machine learning algorithms and PPI network were performed for core gene selection. **A** LASSO regression analysis. **B** SVM-RFE algorithm. **C** Hub genes based on the Degree algorithm in the Cytohubba plugin. **D** Venn diagram showing the overlapping gene of LASSO regression, SVM-RFE algorithm, and hub genes

To explore the biomarkers of asthma, two distinct machine learning algorithms, namely, the LASSO regression and SVM-RFE, were performed. The LASSO regression analysis identified 46 DEGs as signature genes in asthma (Fig. 2A). The SVM-RFE algorithm screened 28 DEGs as characteristic genes in asthma (Fig. 2B). In addition, a PPI network of DEGs was constructed using the STRING database. A core network was then obtained through Degree algorithm in the Cytohubba plugin, and 10 hub genes were identified (Fig. 2C). After combining the results of LASSO regression, SVM-RFE algorithm, and hub genes by Venn diagram, only an intersection gene was identified, i.e., TLR7 (Fig. 2D). TLR7 was thus identified as the core gene for the subsequent research.

### Immune infiltration analyses

**Fig. 3:**
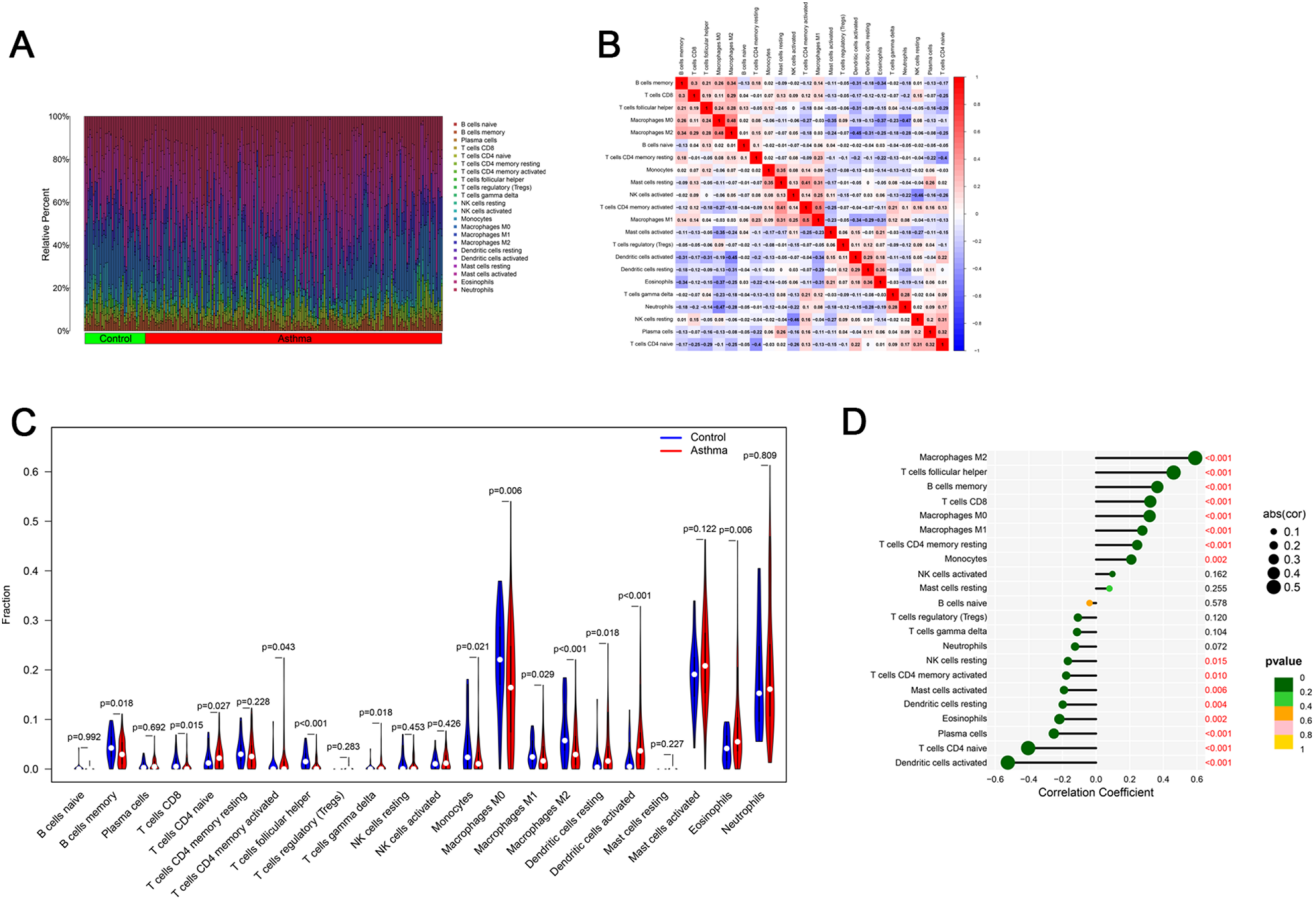
Landscape of immune infiltration between the asthma and healthy control groups. **A** The box plot diagram indicates the relative percentage of different types of immune cells in each sample. **B** The heatmap shows the correlation of infiltrating immune cells. **C** The violin plot shows the differences of immune infiltration between the asthma (red) and healthy control (blue) groups. **D** The lollipop chart presents the correlation of TLR7 and infiltrating immune cells on the basis of Spearman correlation analysis results. (*p* value < 0.05 indicated statistical significance)

CIBERSORT algorithm was used to analyze the difference of immune cell infiltration between the asthma and healthy control groups in 22 subpopulations of immune cells. The total value of all immune cells in each sample was set at 100%, and the proportion of each immune cell in these samples is presented in Fig. 3A. The interaction between the immune cells was also analyzed. Average linkage clustering revealed that M1 macrophages and activated memory CD4 T cells have a significant positive correlation, whereas neutrophils and M0 macrophages are significantly negatively correlated (Fig. 3B). The violin plot showed marked differences in the distribution of 13 out of 22 immune cells (Fig. 3C). The fractions of naive CD4 T cells (*p* = 0.027), resting dendritic cells (*p* = 0.018), activated dendritic cells (*p* < 0.001), and eosinophils (*p* = 0.006) in the asthma group were remarkably higher compared with those of the healthy controls, while the fractions of memory B cells (*p* = 0.018), CD8 T cells (*p* = 0.015), activated memory CD4 T cells (*p* = 0.043), follicular helper T cells (*p* < 0.001), gamma delta T cells (*p* = 0.018), monocytes (*p* = 0.021), M0 macrophages (*p* = 0.006), M1 macrophages (*p* = 0.029), and M2 macrophages (*p* < 0.001) were lower in asthma. Taken together, these results suggest that the heterogeneity of infiltrating immune cells in asthma is evident and may play a role in the pathogenesis of asthma.

To further investigate the correlation of TLR7 and infiltrating immune cells, Spearman correlation was performed (Table 2) and plotted in a lollipop chart (Fig. 3D) and several scatter charts (Additional file 1: Fig S4). The results demonstrated that TLR7 was positively correlated with M2 macrophages (*r* = 0.59, *p* < 0.001), follicular helper T cells (*r* = 0.46, *p* < 0.001), memory B cells (*r* = 0.36, *p* < 0.001), CD8 T cells (*r* = 0.32, *p* < 0.001), M0 macrophages (r = 0.32, *p* < 0.001), M1 macrophages (*r* = 0.28, *p* < 0.001), resting memory CD4 T cells (*r* = 0.24, *p* < 0.001), and monocytes (*r* = 0.21, *p* < 0.01). Meanwhile, TLR7 was negatively correlated with activated dendritic cells (*r* = − 0.52, *p* < 0.001), naive CD4 T cells (*r* = − 0.40, *p* < 0.001), plasma cells (*r* = − 0.25, *p* < 0.001), eosinophils (*r* = − 0.22, *p* < 0.01), resting dendritic cells (*r* = − 0.20, *p* < 0.01), activated mast cells (*r* = − 0.19, *p* < 0.01), activated memory CD4 T cells (*r* = − 0.18, *p* < 0.05), and resting NK cells (*r* = − 0.17, *p* < 0.05). These results indicate that the core gene TLR7 is closely related to the level of immune cell infiltration and plays a crucial role in the immune microenvironment of asthma.

**Table 2:** Correlation of TLR7 and infiltrating immune cells

Infiltrating immune cells	*r*	*p* value
Macrophages M2	0.59	< 0.001
T cells follicular helper	0.46	< 0.001
T cells follicular helper	0.46	< 0.001
B cells memory	0.36	< 0.001
T cells CD8	0.32	< 0.001
Macrophages M0	0.32	< 0.001
Macrophages M1	0.28	< 0.001
T cells CD4 memory resting	0.24	< 0.001
Monocytes	0.21	0.002
NK cells activated	0.10	0.162
Mast cells resting	0.08	0.255
B cells naive	− 0.04	0.578
T cells regulatory (Tregs)	− 0.11	0.120
T cells gamma delta	− 0.11	0.104
Neutrophils	− 0.12	0.072
NK cells resting	− 0.17	0.015
T cells CD4 memory activated	− 0.18	0.010
Mast cells activated	− 0.19	0.006
Dendritic cells resting	− 0.20	0.004
Eosinophils	− 0.22	0.002
Plasma cells	− 0.25	< 0.001
T cells CD4 naive	− 0.40	< 0.001
Dendritic cells activated	− 0.52	< 0.001

### Validation of TLR7 in a GEO dataset and the diagnostic value of TLR7 for asthma

**Fig. 4:**
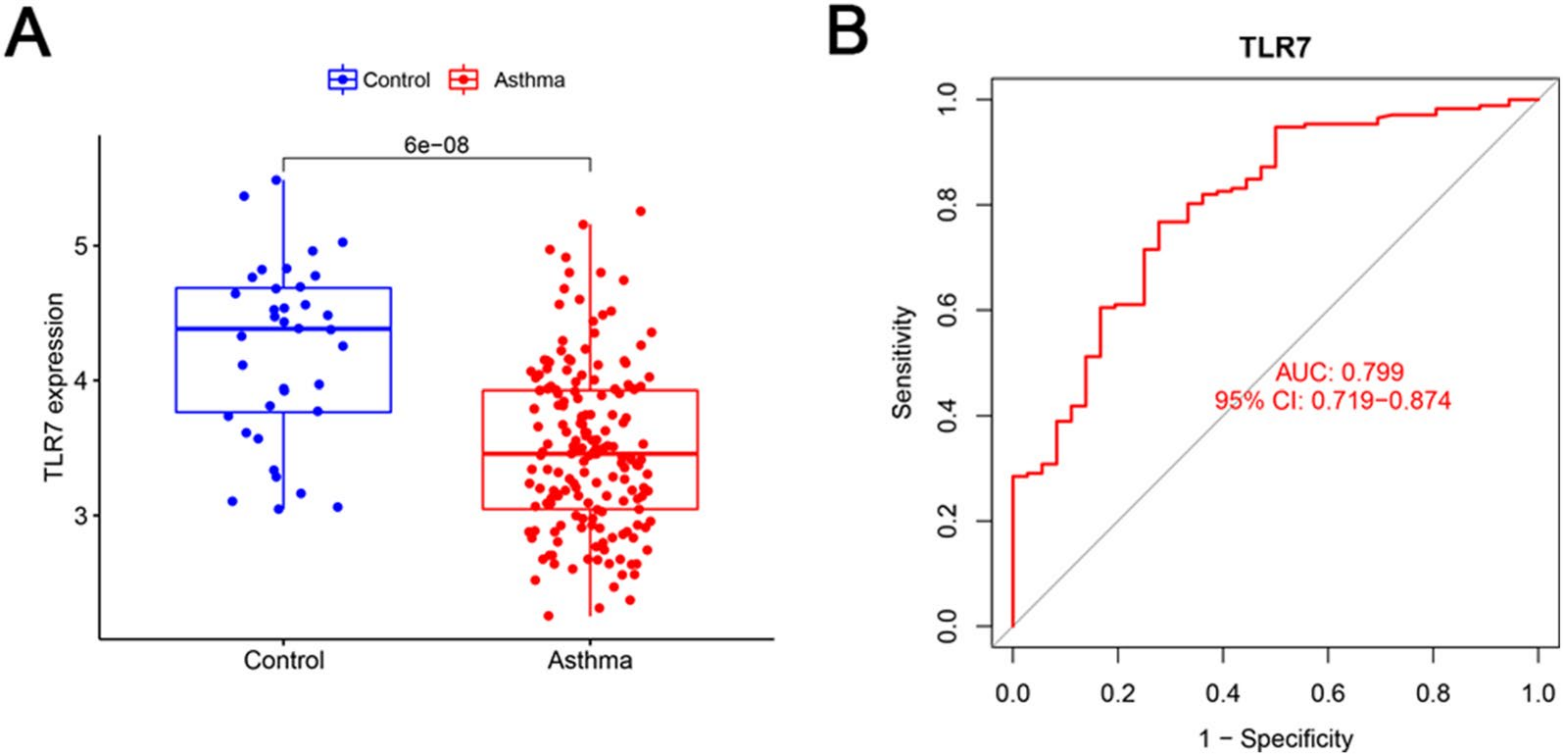
TLR7 expression level and its diagnostic value in asthma. **A** The expression level of TLR7 in the asthma (red) and healthy control (blue) groups in the merged dataset. (*p* value < 0.05 indicated statistical significance). **B** ROC curve analysis of TLR7 in the merged dataset

**Fig. 5:**
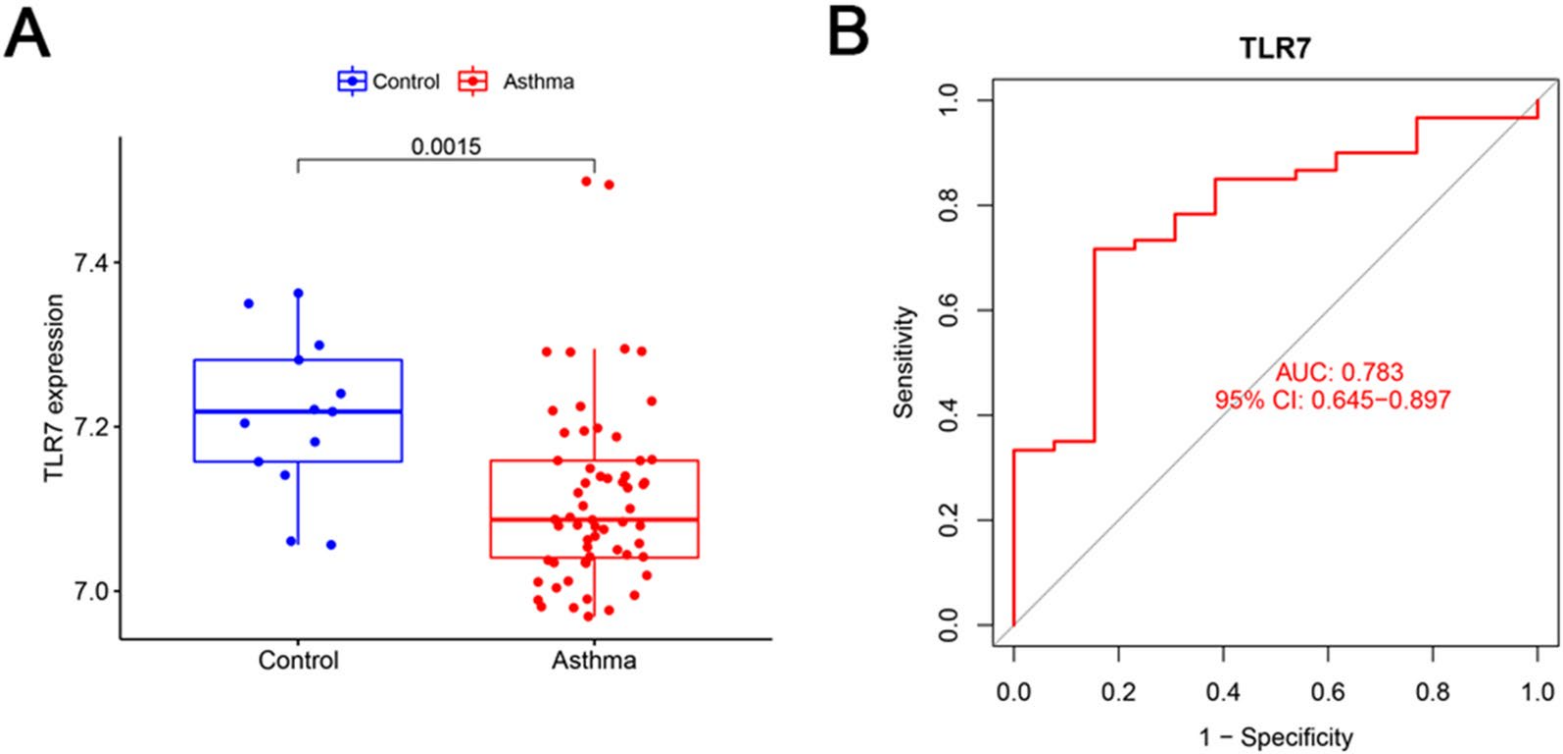
Validation of the expression and diagnostic value of TLR7 in the GSE147878 dataset. **A** The expression level of TLR7 in the asthma (red) and healthy control (blue) groups in the GSE147878 dataset. (*p* value < 0.05 indicated statistical significance). **B** The ROC curve analysis of TLR7 in the GSE147878 dataset

In the merged dataset, the expression level of TLR7 in the asthma group significantly decreased compared with that of the healthy control group (*p* < 0.001, Fig. 4A). ROC curve analysis was conducted to evaluate the sensitivity and specificity of TLR7 for the diagnosis of asthma. As shown in Fig. 4B, the area under curve (AUC) value of TLR7 was 0.799 (95% CI 0.719–0.874). Moreover, the GSE147878 dataset was used to validate the expression and diagnostic effectiveness of TLR7 in asthma. Consistently, the TLR7 expression level in the asthma group of the GSE147878 dataset also significantly decreased (*p* < 0.01, Fig. 5A), and the AUC value of TLR7 was 0.783 (95% CI 0.645–0.897, Fig. 5B).

### Validation of TLR7 mRNA expression in induced sputum cells of asthmatic patients

**Fig. 6:**
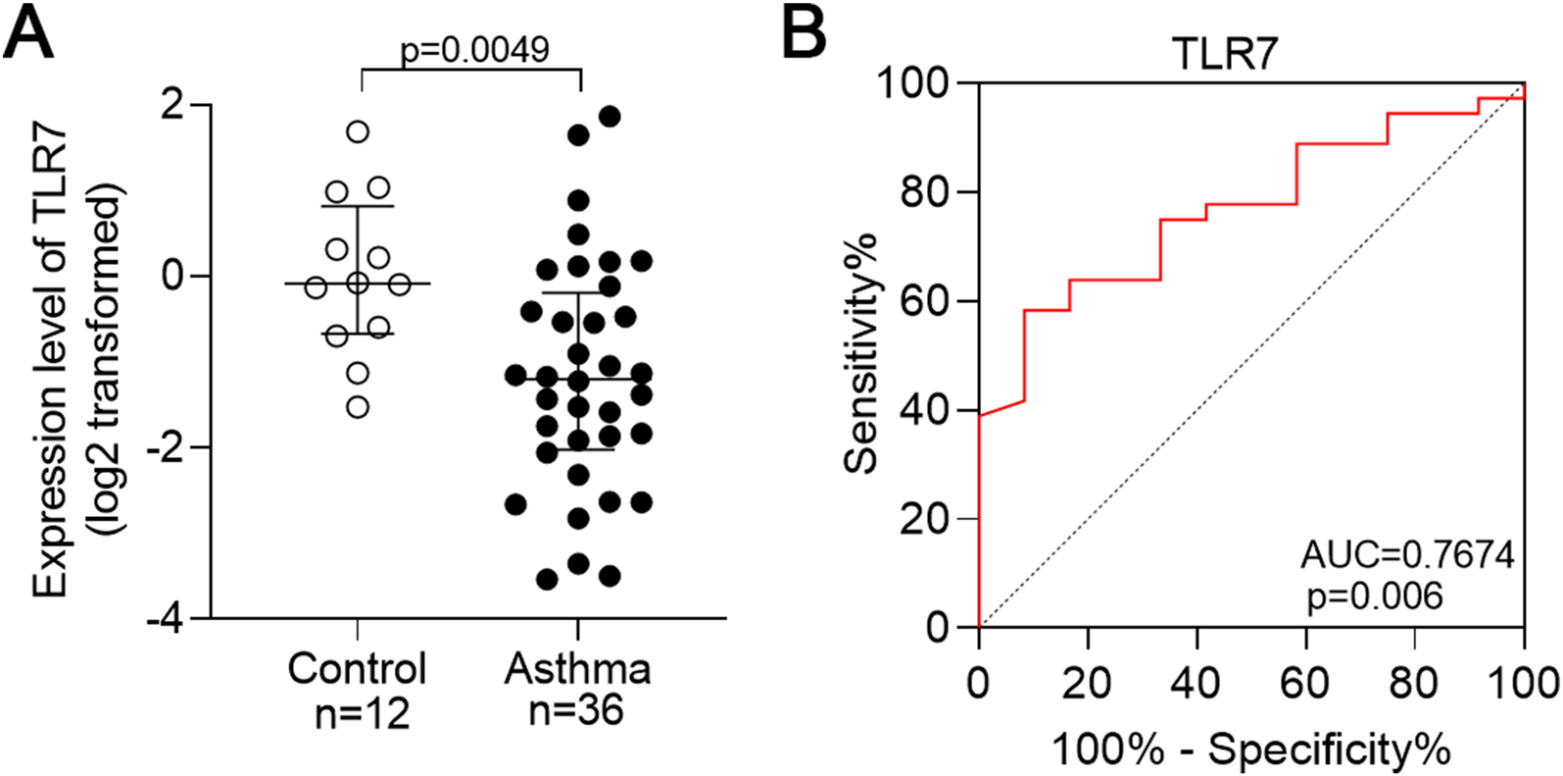
Validation of the expression and diagnostic value of TLR7 in asthmatic patients. **A** TLR7 mRNA expression level in induced sputum cells of asthma. **B** ROC curve of TLR7 in induced sputum cells

Detection of TLR7 mRNA levels via qRT-PCR showed that TLR7 mRNA levels were significantly downregulated in asthmatic patients compared with those in healthy controls (*p* = 0.0049, Fig. 6A). The AUC value was 0.7674 (95% CI 0.631–0.904, *p* = 0.006) (Fig. 6B). Our test results are thus consistent with those of the GEO dataset, and TLR7 has a satisfactory diagnostic ability for asthma.

### TLR7 mRNA expression is associated with airway eosinophilic inflammation and lung function

**Fig. 7:**
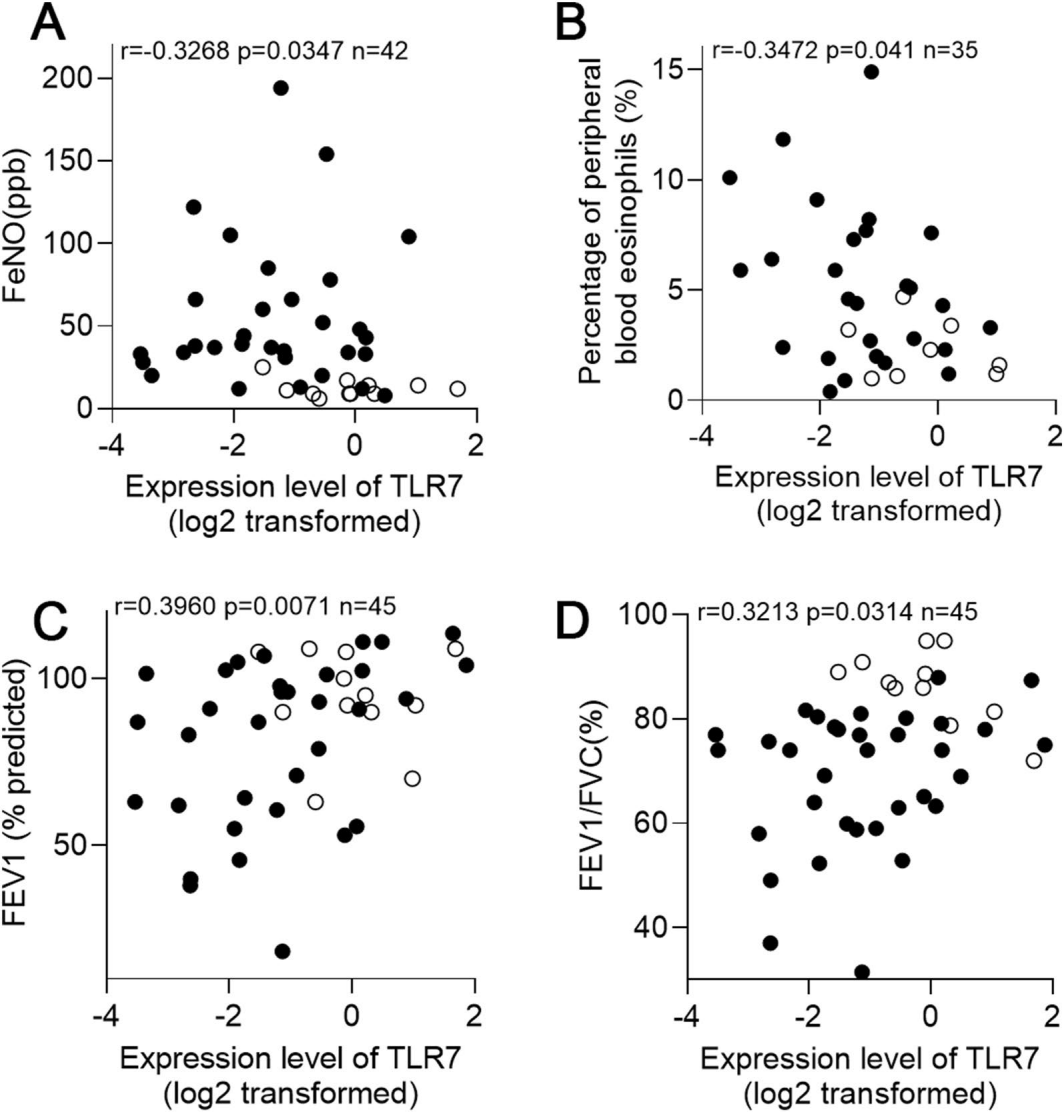
Relationship between TLR7 mRNA expression level and clinical parameters. Relationship between TLR7 mRNA expression level in induced sputum cells and **A** FeNO, **B** percentage of peripheral blood eosinophils (%), **C** FEV1 (% predicted), and **D** FEV1/FVC (%)

We investigated the correlation between TLR7 mRNA expression and clinical indicators such as FeNO, percentage of peripheral blood eosinophils (%), and lung function. The results showed that TLR7 mRNA expression was significantly negatively correlated with FeNO (*r* = − 0.3268, *p* = 0.0347) (Fig. 7A) and percentage of peripheral blood eosinophils (%) (*r* = − 0.3472, *p* = 0.041) (Fig. 7B), and positively correlated with forced expiratory volume in the first second (FEV1) (% predicted) (*r* = 0.3960, *p* = 0.0071) (Fig. 7C) and FEV_1_/forced vital capacity (FVC) (*r* = 0.3213, *p* = 0.0314) (Fig. 7D). These data suggest that TLR7 is involved in the pathogenesis of eosinophilic inflammation and bronchoconstriction in asthmatic patients.

## Discussion

Asthma is a common chronic disease [2]. Induced sputum may have some characteristics as a noninvasive marker of airway inflammation [12]. The gene expression profile of induced sputum cells is altered in patients with asthma [13]. In the current study, two datasets (i.e., GSE76262 and GSE137268), including induced sputum samples of 172 asthmatic patients and 36 healthy controls, were combined for analysis. The combat algorithm in “sva” R package was used to eliminate batch effect [33]. TLR7 was identified as the core gene through the intersection of two different machine learning algorithms (i.e., LASSO regression and SVM-RFE) and the top 10 core networks based on Cytohubba. The immune infiltration analysis results showed that TLR7 is closely related to the level of numerous infiltrating immune cells. Finally, the decreased TLR7 expression levels were validated in induced sputum samples of patients with asthma. The diagnostic value of TLR7 for eosinophilic asthma was evaluated, and its correlation with related clinical indicators was also analyzed.

In the present study, a total of 320 DEGs between the asthma and healthy control groups were obtained. GO and KEGG analyses revealed that DEGs between the asthma and healthy controls were primarily enriched in cytokine–cytokine receptor interaction and immune-related functions, such as immune effector process and immune receptor activity. GSEA is a threshold-free method that analyzes all genes on the basis of their differential expression rank, or other score, without prior gene filtering [34]. GSEA results coincided with the GO and KEGG results. Moreover, these DEGs were proven to be related to lung diseases, such as asthma, by DO analysis. Furthermore, two machine learning algorithms, the LASSO regression and SVM-RFE, were performed to identify the biomarkers of asthma. The combination of LASSO and SVM-RFE has been applied in previous research to identify diagnostic markers [20, 27, 28]. The traditional PPI network of DEGs was also constructed to identify hub genes. After combining the results of LASSO, SVM-RFE, and hub genes, decreased TLR7 was finally identified as the core gene of asthma.

Toll-like receptors (TLRs) play crucial roles in the recognition of invading pathogens and the immune system. The role of TLR signatures in asthma has been reported by Wu et al. that TLR2/TLR3/TLR4 pathway, MyD88-dependent/independent TLR pathway, positive regulation of TLR4 pathway and TLR binding signatures were correlated with asthma [35]. TLR7 is an endosomal receptor that recognizes microbial or self-antigen-derived single-stranded RNA ligands [36]. Currently, TLR7 has been reported to be involved in the pathogenesis of various immunological diseases [37–43]. Research reports that TLR7 agonists reduce Th2-mediated airway inflammation, airway hyperreactivity, and chronic airway remodeling in asthma [44]. Jha A and coworkers also achieved similar results [45]. TLR7 agonists can increase the expression of interferon and C-C motif chemokine ligand 13 (CCL13) in nasal mucosa of patients with asthma and allergic rhinitis [46]. Several research findings also revealed that TLR7 regulates RV1b-induced type I and type III interferon signaling pathways in allergic asthma [47]. TLR7 may confer predisposition to asthma and related atopic diseases [48]. A significant correlation was found between TLR7 single nucleotide polymorphism (SNP) and childhood asthma [49]. Furthermore, the expression of TLR7 in the airway of asthmatic mice was significantly decreased, and upregulation of TLR7 was found to inhibit the activation of NF-κB signaling pathway, reduce airway inflammation, inhibit the proliferation of airway smooth muscle cells (ASMCS), and promote apoptosis in asthmatic mice [50]. Recently, TLR7-nanoparticle adjuvants have been reported to improve the immune response to viral antigens [51]. TLR7 plays a key role in the pathogenesis of rosacea by activating the NFκB-mTORC1 axis [52]. Another study also showed that TLR7 expression is decreased in the lungs of patients with severe asthma [53]. The GSE147878 dataset confirmed that the TLR7 expression level in asthma is also significantly reduced and has good diagnostic value. The expression trend of our test result was consistent the GEO datasets, that is, TLR mRNA expression is significantly decreased in the induced sputum of asthmatic patients and has satisfactory diagnostic ability. TLR7 mRNA expression was significantly negatively correlated with FeNO and percentage of peripheral blood eosinophils (%) and positively correlated with FEV1 (% predicted) and FEV_1_/FVC. We thus inferred that TLR7 is involved in the pathogenesis of eosinophilic inflammation and bronchoconstriction in asthmatic patients.

In addition, immune infiltration analysis in this study demonstrated that the changes of infiltrating immune cells in asthma are evident. Significant differences were observed in the distribution of 13 out of 22 immune cells in asthma. The fractions of dendritic cells and eosinophils in the asthma group were remarkably higher, whereas the fractions of memory B cells, T cells, monocytes, and macrophages were lower compared with those of the healthy controls. Interestingly, TLR7 was also found to be closely related to the level of immune cell infiltration in the current study. Therefore, it could be concluded that TLR7 may play a critical role in asthma by regulating immune cells.

There are also inherent limitations in this study. First, the size of induced sputum samples was not sufficiently large. Further study should include more samples. Second, our sample size was small and we did not compare TLR7 protein levels across different asthma subtypes. Finally, the mechanism by which TLR7 affects eosinophilic asthma was not thoroughly studied. Therefore, further studies are warranted to confirm this mechanism as potential new therapeutic targets of eosinophilic asthma.

## Conclusions

In conclusion, this study proved that decreased TLR7 in the induced sputum of eosinophilic asthmatic patients was involved in immune cell infiltration and airway inflammation, which may serve as a new biomarker for the diagnosis of eosinophilic asthma.

### Supplementary Information

**Additional file 1: Figure S1.** Flow chart of the study design. **Figure S2.** Visualization of differentially expressed genes (DEGs). (A) Heatmap showed the expression of DEGs in each sample. (B) DEGs filtered by thresholds were presented in volcano map. Red dots represent upregulated genes and blue dots represent downregulated genes. **Figure S3.** Functional enrichment analysis of differentially expressed genes (DEGs). (A) GO analysis of DEGs. (B) KEGG analysis of DEGs. **Figure S4.** Scatter charts of the correlation of TLR7 and infiltrating immune cells. Spearman correlation of the correlation of TLR7 and infiltrating immune cells was performed. The results were presented in scatter charts.

## Data Availability

The data that support the findings of this study are available in GEO database (http://www.ncbi.nlm.nih.gov/geo), reference number [GSE76262, GSE137268 and GSE147878].

## Abbreviations

GEO: Gene expression omnibus
DEG: Differential expression gene
TLR7: Toll-like receptor 7
GO: Gene ontology
KEGG: Kyoto Encyclopedia of Genes and Genomes
GSEA: Gene set enrichment analysis
DO: Disease Ontology
LASSO: Least absolute shrinkage and selector operation
SVM-RFE: Support vector machine-recursive feature elimination
PPI: Protein–protein interaction
ROC: Receiver operating characteristic
FeNO: Exhaled nitric oxide fraction
qRT-PCR: Quantitative real‐time polymerase chain reaction
BP: Biological processes
CC: Cellular component
MF: Molecular function
TNF: Tumor necrosis factor
NF: Nuclear factor
JAK/STAT: Janus kinase/signal transducer and activator of transcription
MAPK: Mitogen-activated protein kinase
AUC: Area under curve
FEV1: Forced expiratory volume in the first second
FVC: Forced vital capacity
CCL13: C-C motif chemokine ligand 13
SNP: Single nucleotide polymorphism
