# Supplementary material for: Decreased TLR7 expression was associated with airway eosinophilic inflammation and lung function in asthma: evidence from machine learning approaches and experimental validation
Source: Eur J Med Res. 2024 Feb 10;29:116. doi: 10.1186/s40001-023-01622-5 (PMC10858610; doi:10.1186/s40001-023-01622-5)
Supplement: Supplementary file 1 — Additional file 1: Figure S1. Flow chart of the study design. Figure S2. Visualization of differentially expressed genes (DEGs). (A) Heatmap showed the expression of DEGs in each sample. (B) DEGs filtered by thresholds were presented in volcano map. Red dots represent upregulated genes and blue dots represent downregulated genes. Figure S3. Functional enrichment analysis of differentially expressed genes (DEGs). (A) GO analysis of DEGs. (B) KEGG analysis of DEGs. Figure S4. Scatter charts of the correlation of TLR7 and infiltrating immune cells. Spearman correlation of the correlation of TLR7 and infiltrating immune cells was performed. The results were presented in scatter charts. [file 40001_2023_1622_MOESM1_ESM.docx]

**Supplementary Figures**

**
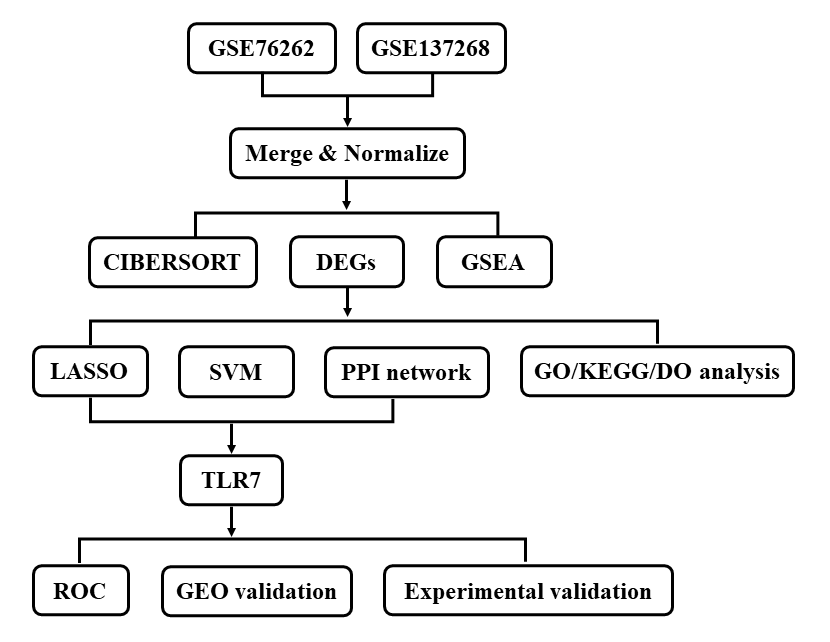
**

**Supplementary Figure 1: Flow chart of the study design.**

**
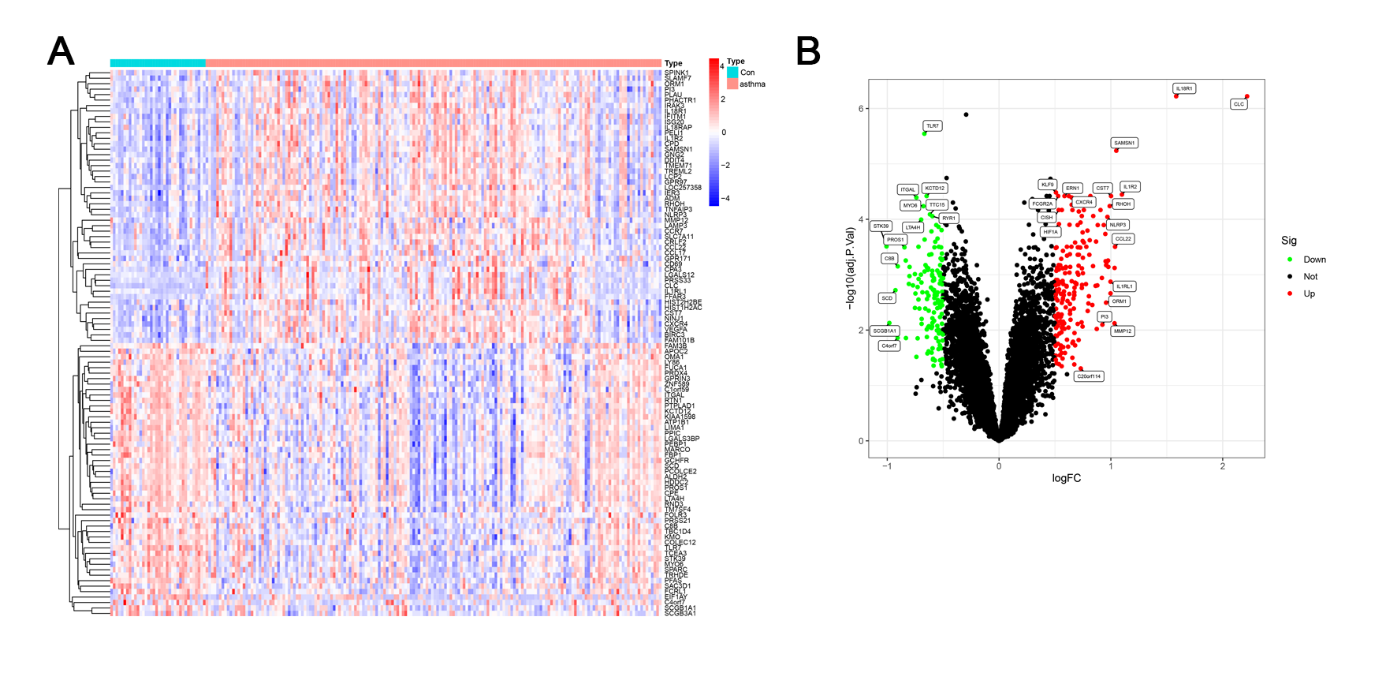
**

**Supplementary Figure 2: Visualization of differentially expressed genes (DEGs).**

**
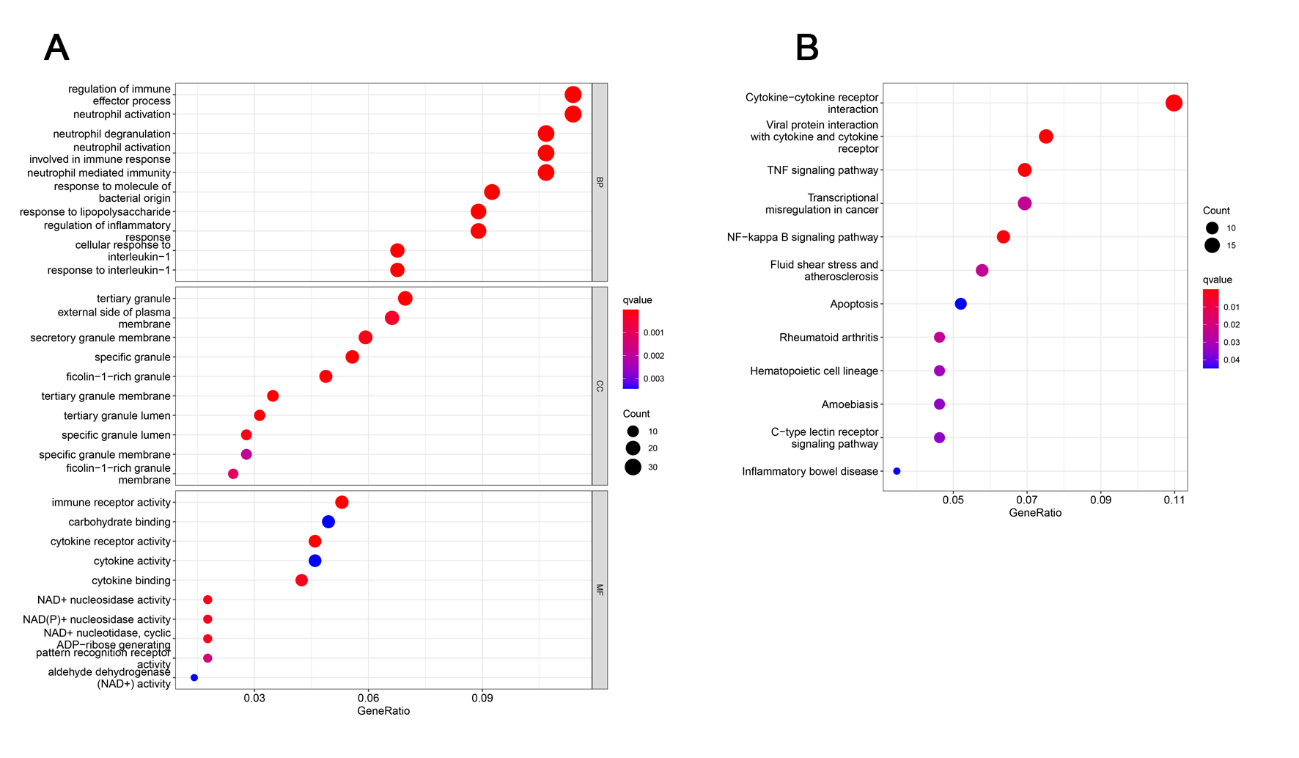
**

**Supplementary Figure 3: Functional enrichment analysis of differentially expressed genes (DEGs).**

**
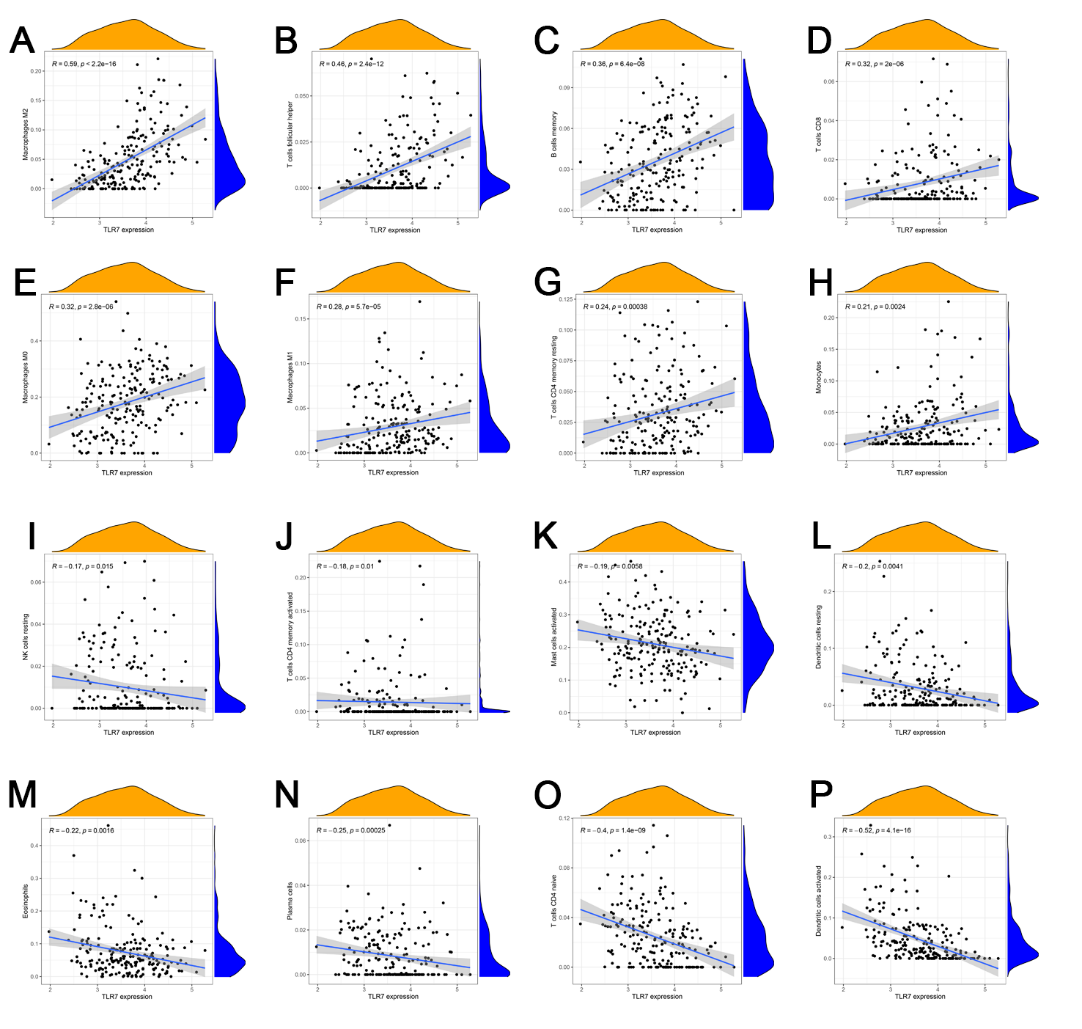
**

**Supplementary Figure 4: Scatter charts of the correlation of TLR7 and infiltrating immune cells.**

**Supplementary Figure Legends:**

**Supplementary Figure 1: Flow chart of the study design.**

**Supplementary Figure 2: Visualization of differentially expressed genes (DEGs).** (A) Heatmap showed the expression of DEGs in each sample. (B) DEGs filtered by thresholds were presented in volcano map. Red dots represent up-regulated genes and blue dots represent down-regulated genes.

**Supplementary Figure 3: Functional enrichment analysis of differentially expressed genes (DEGs).** (A) GO analysis of DEGs. (B) KEGG analysis of DEGs.

**Supplementary Figure 4: Scatter charts of the correlation of TLR7 and infiltrating immune cells.** Spearman correlation of the correlation of TLR7 and infiltrating immune cells was performed. The results were presented in scatter charts.
